# Adherence to the Chinese or American Dietary Guidelines is Associated with a Lower Risk of Primary Liver Cancer in China: A Case-Control Study

**DOI:** 10.3390/nu10081113

**Published:** 2018-08-17

**Authors:** Pei-Yan Chen, Ai-Ping Fang, Xiao-Yan Wang, Qiu-Ye Lan, Gong-Cheng Liao, Zhao-Yan Liu, Dao-Ming Zhang, Yao-Yun Zhang, Yu-Ming Chen, Hui-Lian Zhu

**Affiliations:** 1 Department of Nutrition, School of Public Health, Sun Yat-sen University, Guangzhou 510080, China; chpyan@mail2.sysu.edu.cn (P.-Y.C.); fangaip@mail.sysu.edu.cn (A.-P.F.); wangxy277@mail2.sysu.edu.cn (X.-Y.W.); bolivia.lan@gmail.com (Q.-Y.L.); liaogch3@mail2.sysu.edu.cn (G.-C.L.); liuzhy69@mail2.sysu.edu.cn (Z.-Y.L.); zhangdm3@mail2.sysu.edu.cn (D.-M.Z.);; 2 Department of Hepatobiliary Surgery, Sun Yat-sen University Cancer Center, Guangzhou 510060, China; zhangyuj@sysucc.org.cn; 3 Guangdong Provincial Key Laboratory of Food, Nutrition and Health; Guangzhou 510080, China; chenyum@mail.sysu.edu.cn; 4 Department of Medical Statistics & Epidemiology, School of Public Health, Sun Yat-sen University, Guangzhou 510080, China

**Keywords:** dietary index, diet, liver cancer, risk factors, case-control study

## Abstract

Adherence to healthy dietary guidelines has been related to a lower risk of several cancers, but its role in primary liver cancer (PLC) has not been fully investigated, especially among Eastern populations. This study enrolled 720 PLC patients and 720 healthy controls who were frequency-matched by age and sex between September 2013 and October 2017 in South China. Dietary quality was assessed by the Chinese Healthy Eating Index (CHEI) and the Healthy Eating Index 2015 (HEI-2015), which manifests as scores of adhering to the 2016 Dietary Guidelines for Chinese and adhering to the 2015–2020 Dietary Guidelines for Americans, respectively. Odds ratios (ORs) and 95% confidence intervals (CIs) were estimated using unconditional logistic regression models, adjusting for potential confounders. Higher scores in both the CHEI and HEI-2015 were associated with a lower risk of PLC (per 5-points increment of the total scores: OR: 0.43, 95% CI: 0.38–0.50 for CHEI; OR: 0.47, 95% CI: 0.40–0.55 for HEI-2015). The protective associations persisted significantly in the stratified analyses by sex, smoker status, alcohol consumption, HBV infection, and histological types of PLC, without statistical evidence for heterogeneity (*p*-interaction > 0.05). Closer adherence to the most recent dietary guidelines for Chinese or Americans may protect against PLC.

## 1. Introduction

Primary liver cancer (PLC), one of the most malignant cancers with a poor prognosis, was the sixth most common cancer, and the second leading cause of cancer-related death worldwide in 2012. Almost half of the total number of cases and deaths occurred in China alone [1]. Although most PLC cases are attributable to chronic infection with viral hepatitis (Hepatitis B virus (HBV) or Hepatitis C virus (HCV)), other risk factors have been identified, including heavy alcohol consumption, tobacco smoking, exposure to aflatoxins, obesity and diabetes [2]. Additionally, accumulating evidence suggests that dietary factors may also be relevant to the development of PLC. Epidemiological studies have shown that a higher intake of vegetables [3], white meat [4], dairy [5], and soy foods [6] as well as a lower intake of red meat and saturated fat [7] were associated with the reduced risk of PLC. 

Given that foods are consumed in combination, and are highly correlated with each other, it is difficult to distinguish their individual effects [8,9]. Dietary pattern analysis, considering overall diet rather than a single food or nutrient, can overcome this challenge. Two approaches, the ‘posteriori’ (data driven) approach and the ‘priori’ (index-based) approach, are used to derive dietary patterns. The former generates dietary patterns empirically in a population but may vary in another population. The latter measures adherence to established dietary guidelines and is easier to reproduce and more comparable across studies [10].

Adherence to healthy eating guidelines has been related to a lower risk of several cancers, e.g., breast cancer [11,12], colorectal cancer [13], pancreatic cancer [14], esophageal cancer [15] and nasopharyngeal cancer [16], but its role in primary liver cancer (PLC) has not been fully investigated. To our knowledge, few studies have reported the association of index-based dietary patterns with PLC incidence. Existing data have focused on the traditional Mediterranean diet scores in Italy and Greece [17], as well as the alternate Mediterranean diet scores and the Healthy Eating Index-2010 (HEI-2010) in America [18]. However, information is still scarce regarding Eastern populations, with different dietary and lifestyle patterns and a higher prevalence of HBV infection. In the current study, we comprehensively evaluated associations between two diet quality indexes, the Chinese Healthy Eating Index (CHEI), which reflects the 2016 Dietary Guidelines for Chinese [19], and the most recent version of the HEI (HEI-2015), which aligns with the 2015-2020 Dietary Guidelines for Americans [20], and the risk of developing PLC in a large case-control study undertaken in China.

## 2. Materials and Methods 

### 2.1. Study Population

This case-control study was conducted from September 2013 to October 2017. PLC patients were recruited at the Sun Yat-sen University Cancer Center. Ascertainment of the PLC diagnosis was based on biopsy, computed tomography (CT), magnetic resonance imaging (MRI), or elevated a-fetoprotein (AFP) levels, according to the National Comprehensive Cancer Network (NCCN) Clinical Practice Guidelines in Oncology: Hepatobiliary Cancers [21]. Patients aged 18–80 years were eligible for inclusion in this study if they were newly diagnosed within one month, were previously untreated, had been residents of Guangdong Province for at least five years or had eaten similar cuisine. Controls were healthy adults with no prior history of PLC who were recruited via advertisements, flyers or subjects’ referrals in the same time period from communities in the same province as the patients. Patients and controls with any of the following conditions were excluded: (1)had a history of other malignant cancers (63 cases); (2) had substantial changes in dietary habits due to severe chronic diseases or intention to control diet mainly according to doctor’s advice within the past five years (77 cases and 75 controls); (3) had implausible daily energy intake values (i.e., <700 or >4200 kcal for men, <500 or >3500 kcal for women) or an incomplete dietary assessment (80 cases and 2 controls); (4) had no blood samples for HBV tests (10 controls); and (5) refused to participate in the study (158 cases). The patients and controls were frequency-matched by age (±5 years) and sex. Finally, 720 patients and 720 controls were included in the present analysis.

This study protocol was approved by the Ethics Committee of the School of Public Health at Sun Yat-sen University. Written informed consent was obtained from all participants.

### 2.2. Data Collection

All eligible participants provided detailed information on sociodemographic characteristics (e.g., age, sex, marital status, education level, and household monthly income per capita), lifestyle habits (e.g., smoking, alcohol and tea drinking), and daily physical activity over the past 12 months before diagnosis for patients as well as before the interview for controls, and personal medical history of selected diseases (e.g., hypertension, diabetes, heart diseases, and renal diseases) was obtained via face-to-face interviews. Current smokers or alcohol drinkers were defined as subjects who had been smoking at least one cigarette per day or drinking alcohol beverages once a week for at least six months. Former smokers were once smokers but had quit smoking at least one year prior. Low and high intakes of alcohol beverages were defined by alcohol consumption of 1–15 g/d and over 15 g/day in this study. Individuals were considered as tea drinkers if they drank tea at least twice every week during the last year prior to the interview. Physical activity was evaluated using a 19-item questionnaire by summing the products of the time spent on a variety of activities, including sleeping, working, transportation, housework, leisure, and exercises, with the mean metabolic equivalent (MET) for that activity [22]. Body height and weight were measured following standard protocols. Body mass index (BMI) was then calculated as weight (kg) divided by height squared (m^2^). Diagnostic data were obtained from the electronic clinical and administrative system of the Sun Yat-sen University Cancer Center. The histological types of PLC included hepatocellular carcinoma (HCC), intrahepatic cholangiocarcinoma (ICC), and mixed HCC and ICC. 

Fasting blood samples were collected from the patients and controls at recruitment. Seropositivity of hepatitis B surface antigen (HBsAg) was measured by using an enzyme-linked immunosorbent assay (ELISA; Diagnostic Kit for Hepatitis B Virus Surface Antigen, Shanghai Kehua Bio-Engineering Co., Ltd., Shanghai, China).

### 2.3. Dietary Assessment

Usual dietary intake during the preceding year before PLC diagnosis for the patients or before the interview for controls was assessed by using a 79-item semiquantitative food-frequency questionnaire (FFQ). The FFQ had been validated through six nonconsecutive, 3-day, dietary records; the energy-adjusted correlation coefficients for nutrients and food groups ranged from 0.46 to 0.71 and 0.36 to 0.66, respectively [23]. The participants were asked about the frequency and potion size of each food consumed, according to 5 predefined response categories (never, yearly, monthly, weekly, or daily) and 2 predefined potion sizes (1 Liang = 50 g, or 1 cup = 200 ml). A standard set of pictures was used to help participants quantify their consumption. Average daily intakes of nutrients and total energy were calculated by multiplying the daily intake of each food item by the corresponding nutrient content obtained from the China Food Composition Table 2009 [24]. 

We transformed daily food and nutrient intakes into standard potions based on the 2016 Dietary Guidelines for the Chinese and the 2013-2014 Food Patterns Equivalents Database, respectively [19,25]. Given that the information on salt consumption, the main food source of sodium, was not collected in the FFQ, we assigned a score ranging from 0 to 10 by 2.5 to reflect an individual’s sodium intake according to taste preference (i.e., very salty, salty, moderate, mild, very mild). 

#### 2.3.1. Chinese Healthy Eating Index

The CHEI was designed to assess adherence to the 2016 Dietary Guidelines for the Chinese, the most current and scientific nutritional guideline for the Chinese [19]. The total scores of the CHEI range from 0 (nonadherence) to 100 (perfect adherence). The CHEI consists of 17 components: 14 components (total grains, whole grains and mixed beans, tubers, total vegetables, dark vegetables, dairy, soybeans, fish and seafood, poultry, eggs, seeds and nuts, red meat, added sugars, and alcohol) are worth 0 to 5 points each, and the remaining 3 components (fruits, cooking oils, and sodium) are worth 0 to 10 points each [19]. Red meat, cooking oils, sodium, added sugars, and alcohol are five limitation components, in which a higher intake indicates a lower score. The remaining twelve foods are adequacy components, and higher consumption indicate higher score. For these items, zero intake and recommended intake or above would respectively get a zero point or full point (5 or 10 points), while intermediate intakes between zero and the relevant recommended value would award a score according to the formula (score = [actual intake/recommended intake] × full point). All food components are energy-adjusted on a density basis (per 1000 calories), except for sugar (percentage of energy) and alcohol (absolute intakes). Alcohol consumption of no more than 25 g/day for men and no more than 15 g/day for women is awarded 5 points, whereas more than 60 g/day for men and more than 40 g/day for women are awarded 0 points. A higher score means a better adherence to the Dietary Guidelines for the Chinese. Details of this are listed in Supplemental Table S1.

#### 2.3.2. Healthy Eating Index-2015

The HEI-2015 was designed to evaluate concordance with the 2015–2020 Dietary Guidelines for Americans [20]. The index contains 13 components, with total scores ranging from 0 (nonadherence) to 100 (optimal adherence). Six components (total fruits, whole fruits, total vegetables, greens and beans, total protein foods, and seafood and plant proteins) are awarded 0 to 5 points each; the other seven components (whole grains, dairy, fatty acids, refined grains, sodium, added sugars, and saturated fats) are awarded 0 to 10 points each. Scores are calculated proportionately according to the intakes between the minimum and maximum standards. Components are determined by using the density per 1000 calories to account for differences in energy intake among individuals. In this most recent version of the HEI, alcohol contributes extra calories to total energy rather than being used as a single component. The major features of HEI-2010 are reserved, while a small change in scoring for legumes has been made, which is allocated to four components, including total protein foods, seafood and plant proteins, total vegetables, and greens and beans. Four components (refined grains, sodium, added sugars and saturated fats) are moderated (higher intakes receive lower scores). The code used for calculating scores of HEI-2015 was developed by the Division of Cancer Control and Population Sciences in the National Cancer Institute [26].

### 2.4. Statistical Analysis

Differences in characteristics between the patients and controls were compared by using the *t* test or Wilcoxon signed ranks test for continuous variables as well as the chi-square test for categorical variables. Spearman’s correlation coefficients were calculated between the two indexes. To gain insight into the distributions of food intake, we divided the scores of each component of the CHEI and HEI-2015 into four groups, which were 0, (0–50% of maximum score), [50% of maximum score- maximum score), and maximum score. The four groups were classified according to the range of percentage of full scores ([individual score/full score] × 100%): 0.0% (0 point), 0.1–49.9%, 50–99.9% and 100% (full points). For the full points of 5 and 10, the cutoffs were 0.0, 0.1–2.4, 2.5–4.9 and 5.0, and 0.0, 0.1–4.9, 5–9.9 and 10.0, respectively. We compared the proportion of participants in the four groups between the PLC patients and controls by the chi-square test. Odds ratios (ORs) and the corresponding 95% confidence intervals (95% CIs) were estimated in logistic regression models, and the scores served as continuous variables (per 5-point increments). We conducted an alternative analysis by comparing the risk of PLC in the participants with lower adherence (quartile 1 vs. combined quartile 2 to quartile 4 of CHEI/HEI-2015). Regression models were adjusted for age, sex, BMI, physical activity (MET-h/day), education level (secondary school or below, high school or above), household monthly income per capita (≤¥1500, ¥1501-3000, ≥¥3001), smoker status (current, former or never), alcohol consumption (no intake, low intake or high intake), history of diabetes (non-diabetes, diabetes or missing), HBV seropositivity (yes or no), and non-alcohol energy intake (kcal/day). To avoid misclassifying diabetes due to missing values (11 cases and 13 controls), we classified the history of diabetes status into three nominal categories (0 = non-diabetes, 1 = diabetes, 2 = missing) in the multivariable logistic regression analysis. We replaced non-alcohol energy intake with total energy intake in the models for HEI-2015, and the retained results were similar. To explore potential effect modification, we included interaction terms formed by the product of modifying variable categories and the continuous scores. Stratified analyses were then conducted by sex, smoker status, alcohol consumption, HBV infection status, and histological types (HCC or not). We also investigated the independent association between the specific component scores (1-point increment) and the risk of PLC in the multivariable model. Two-sided *p* < 0.05 were considered statistically significant. All statistical analyses were performed using SPSS version 24.0 (SPSS, Chicago, IL, USA).

## 3. Results

### 3.1. Characteristics of the Participants

The general characteristics of the patients and controls are presented in Table 1. The patients and controls were matched by age and sex. The mean ages of the patients and controls were 58.2 (SD: 8.8) years and 58.4 (8.1) years, respectively, and 85.1% were men. Among the 720 PLC patients, 93.6% were diagnosed with HCC. Compared with the controls, the patients were more likely to have higher household income, be infected with HBV, be married, and be current smokers and alcohol drinkers but were less likely to have a higher BMI, physical activity, educational level and total energy intake. The mean scores for the patients and controls were 51.92 (10.31) and 63.95 (7.58) for CHEI and 56.93 (6.68) and 61.43 (5.60) for HEI-2015, respectively. The CHEI and HEI-2015 scores were significantly correlated (Spearman’s r = 0.73; *p* < 0.0001).

### 3.2. Percentage Distribution of the Participants for Each Component

**Figure 1:**
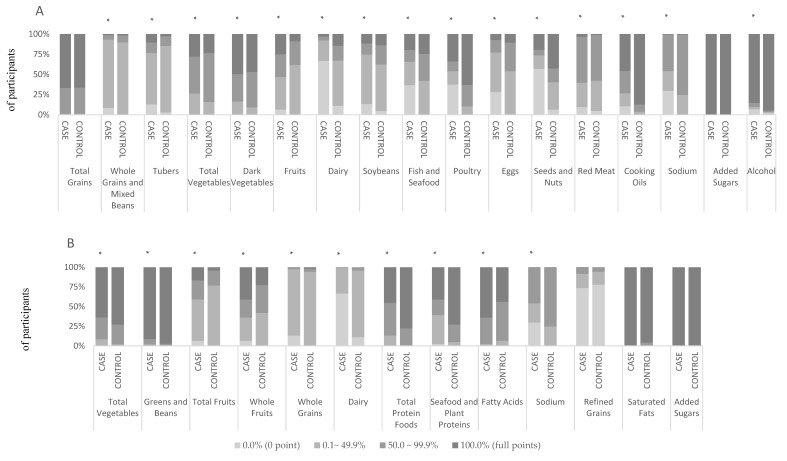
Comparison of the percentage distribution of the cases and controls according to the scores of each item for the CHEI (A) and the HEI-2015 (B). For fruits, sodium and cooking oils in the CHEI and whole grains, dairy, fatty acids, refined grains, sodium, added sugars and saturated fats in the HEI-2015, the cutoffs for the four groups are 0.0, 0.1–4.9, 5.0–9.9 and 10.0. For the remaining components in the CHEI and the HEI-2015, the cutoffs for the four groups are 0.0, 0.1–2.4, 2.5–4.9 and 5.0. *: *p* < 0.01 (chi-square test).

The score distributions of the patients and controls for each component of the CHEI and the HEI-2015 are shown in Figure 1. In general, more than 60% of the patients and controls reached the recommendations for total grains, added sugars, and alcohol in the CHEI; and for total vegetables, greens and beans, added sugars, and saturated fats in the HEI-2015. Most of the PLC patients and controls. However, they did not meet 50% of the recommendations for whole grains and mixed beans (93.1%), tubers (76.5%), dairy (92.0%), soybeans (74.6%), and eggs (87.1%) in the CHEI, or the recommendations for total fruits, whole grains, dairy, and refined grains in the HEI-2015. It is notable that a higher proportion of patients than controls showed consumption of dairy, soybeans, fish and seafood, poultry, eggs, seeds and nuts, cooking oils, and sodium in the CHEI—below 50% of the recommendations for the Chinese. In addition, a higher proportion of patients than controls received zero scores for whole grains and mixed beans, tubers, fruits, dairy, soybeans, fish and seafood, poultry, eggs, seeds and nuts, red meat, cooking oils, sodium, and alcohol in the CHEI, as well as total fruits, whole fruits, whole grains, dairy, seafood and plant proteins, and sodium in the HEI-2015. 

### 3.3. Association of CHEI and HEI-2015 with Primary Liver Cancer Risk

Significantly inverse associations were observed between the CHEI and HEI-2015 scores (considered as continuous variables, per 5-point increments) and PLC risk (OR, 0.43; 95% CI: 0.38–0.50 for CHEI; OR, 0.47; 95% CI: 0.40–0.55 for HEI-2015) in a multivariable adjusted model. The ORs (95%CIs) of PLC for the group with lower adherence (the lowest quartile of CHEI/HEI-2015) compared with the higher adherence group (combined quartiles 2 to 4 [served as reference]) in multivariable adjusted model were 9.31 (6.12, 14.17) and 4.05 (2.75, 5.97), respectively. Similar associations were found across all subgroups stratified by sex, smoker status, alcohol consumption, HBV infection status, and histological types (all *p*-interaction > 0.05) (Table 2). 

### 3.4. Association of Components in CHEI and HEI-2015 with Primary Liver Cancer Risk 

The contribution of each component score in the CHEI and HEI-2015 are shown in Table 3. Among the adequacy components, in which a higher intake received a higher score, eight of the CHEI (whole grains and mixed beans, vegetables, dairy, soybeans, fish and seafood, poultry, eggs, and seeds and nuts), and six of the HEI-2015 (vegetables, greens and beans, whole grains, dairy, total protein foods and seafood and plant proteins) showed significantly inverse associations with PLC risk. The total fruits (including fruit juice) component of the two dietary indexes increased the PLC risk (OR, 1.09; 95% CI 1.03–1.15 for CHEI; OR, 1.25; 95% CI 1.11–1.41 for HEI-2015), but the whole fruits (except for fruit juice) component in the HEI-2015 was not associated with PLC risk (OR, 1.11; 95% CI 1.00–1.23). Among the limitation components, in which a lower intake received a higher score, three of the CHEI (cooking oil, sodium and alcohol) as well as sodium in the HEI-2015 were associated with the reduced risk of PLC, but the fatty acids may increase PLC risk (OR, 1.33; 95% CI 1.20–1.47). The scores in the components of added sugars in both indexes and saturated fats in the HEI-2015 were similar between the patients and controls, which made it difficult to explore their effects.

## 4. Discussion

In this large case-control study, with 720 patients and 720 control subjects conducted in China, we found that a higher score in the CHEI or HEI-2015, reflecting better adherence to the most recent and authoritative dietary guidelines for the Chinese or Americans, was independently associated with a decreased risk of PLC. 

Until now, few studies have investigated the association between diet quality and the risk of developing PLC [17,18,27]. A combined case-control study undertaken in Italy and Greece reported a protective effect of compliance with the traditional Mediterranean diet on HCC [17]. Similarly, in the National Institutes of Health -AARP Diet and Health (NIH-AARP) study, a large U.S. prospective cohort study, the HEI-2010 and the alternate Mediterranean Diet Score (aMED) were reported to be inversely associated with HCC incidence and chronic liver disease (CLD) mortality [18]. In addition, closer adherence to the Dietary Guidelines for Americans or the Mediterranean diet has been reported to protect against nonalcoholic fatty liver disease (NAFLD) [28], obesity [29] and diabetes [30], all of which are well-defined risk factors for PLC [31]. Consistent with these findings from previous studies, our findings suggest a beneficial role of improving diet quality in the prevention of PLC. We also found the participants with lower adherence (Q_1_ vs Q_2-4_) had higher risk of PLC.

Although different approaches are used to award optimal scores in the MED, aMED, HEI-2010, HEI-2015, and CHEI, all these healthy eating diets are characterized by a high intake of vegetables, fruits, whole grains, legumes, seeds and nuts, fish and seafood, lean poultry, and dairy products, as well as a moderate intake of alcohol and a low intake of red meat, cooking oils, and added sugars. Using a data-driven approach, a prospective study from the Shanghai Men’s and Women’s Health Studies showed that a vegetable-based dietary pattern was related to reduced liver cancer risk, whereas fruit- and meat-based dietary patterns showed no association [27]. This observation was in accordance with a meta-analysis on the association between consumption of vegetables and fruits and HCC risk; an increased intake of vegetables but not fruits was linked to a reduced risk of HCC [32]. However, a detrimental effect of the fruit components in the HEI-2010 and aMED on HCC has been reported by Li and colleagues [18]. In our study, the total fruit component (including whole fruits and fruit beverages) and fruit beverages (OR, 1.78; 95% CI 1.36-2.32), but not whole fruit, increased the PLC risk. This discrepancy might be a result of increased fructose consumption from fruit beverages that were associated with NAFLD and fibrosis [33,34]. Therefore, these results should be interpreted with caution and need to be investigated in further prospective studies. In addition, the results from the available data indicate that a higher consumption of fish, white meat, and grains and a lower intake of red meat and dietary sugar were associated with the reduced risk of HCC [4,7,35]. Furthermore, healthy diets are often rich in antioxidants (e.g., flavonoids, beta-carotene, vitamin C, vitamin D, and selenium), fiber, and unsaturated fatty acids, which have been observed to protect against HCC [4,36,37,38]. Additionally, the ratio of monounsaturated to saturated fat in the aMED and the ratio of total poly- and monounsaturated to saturated fatty acids in the HEI-2010 also showed inverse associations with HCC incidence [18]. For our results, the cooking oil component in the CHEI was also inversely associated with PLC risk (OR: 0.71; 95% CI 0.65–0.77), whereas the ratio of total poly- and monounsaturated to saturated fatty acids in HEI-2015 showed an inverse association (OR: 1.33; 95% CI 1.20–1.47). Although the reason for this finding is unclear, it may be that the increased unsaturated acid intake in the present study participants comes from the high consumption of plant cooking oils, mainly containing n-6 fatty acids, which may not be beneficial in HCC [39].

It is well-known that oxidative stress and chronic inflammation induced by exposure to toxic compounds or the hepatitis virus play an important role in the development of PLC [2,40]. Diet quality has been inversely correlated with inflammatory biomarker levels and free radical production [41,42,43,44]. Diet could potentially modify inflammation and thus affect liver carcinogenesis. A case-control study from Italy indicated that a proinflammatory diet, also sharing similar compositions to unhealthy diets, was associated with an increased risk of HCC [45]. Moreover, dietary fiber from whole grains, legumes and vegetables may improve insulin sensitivity due to its low GI or GL and anticholesterolemic action [35,46]. Conversely, saturated fatty acids and dietary heme iron mainly sourced from red meat may promote hepatocarcinogenesis through affecting lipid profiles in the liver and accelerating hepatocyte injury and death [7,47]. 

In stratified analyses, we found that the favorable associations between the two indexes and PLC risk persisted across strata of sex, smoker status, alcohol consumption, HBV infection, and histological types of PLC, with no statistical evidence for heterogeneity. Considering that alcohol is an important risk factor for the etiology of PLC, we adjusted for alcohol consumption (no intake, low intake, or high intake) in the multivariate analysis. Furthermore, we evaluated the scores after removing alcohol consumption in categories, and the results were similar. Taken together, these findings indicate that diet is a risk factor for the development of PLC, independent of other well-known risk factors, e.g., smoker status, alcohol consumption, and HBV infection. 

Our study has several strengths. To our knowledge, this is the first study to investigate the association between the two newest dietary indexes, the CHEI and the HEI-2015, and the risk of PLC. Furthermore, the sample size was large, and the patients and controls were matched by age and sex, thus increasing the statistical power. All the patients were confirmed as PLC patients by morphology and histology, thereby reducing disease misclassification. Additionally, information on HBV infection status in the patients and controls was available, as well as other risk factors, including smoker status, alcohol consumption, obesity, and diabetes. Finally, participants were blinded to the hypothesis, thus reducing information bias.

Several limitations in our study also warrant mention. Firstly, we cannot rule out the possibility of reverse causality with a case-control design. To minimize any possible bias in dietary modification, participants were excluded if they had changed their dietary habits in the previous five years. Secondly, recall bias is inevitable in a retrospective study. To reduce this bias, only incident PLC patients diagnosed within 1 month were enrolled and interviewed as soon as possible after diagnosis. In addition, we provided photographs to help participants quantify habitual intake of foods and beverages consumed during the face-to-face interview. Thirdly, the information provided by general healthy controls did not have perfect comparability with that provided by hospital patients. To control this bias, we selected patients who had resided in Guangdong province for at least 5 years or had eaten similar cuisine to Cantonese cuisine, and, in a sensitivity analysis, after excluding patients with non-Cantonese cuisine (*N* = 84), the association of CHEI or HEI-2015 with PLC risk did not change, and the ORs (95% CI) remained similar (data not shown). Fourthly, priori dietary indexes were derived based on current knowledge, and the CHEI and HEI-2015 were not specifically designed for the prevention of PLC. Moreover, this approach may have limitations when the dietary intake of a component does not vary considerably in a population, such as alcohol, added sugars in the CHEI as well as saturated fats and added sugars in the HEI-2015 in our study, and the same scores did not necessarily reflect similar diets since different individual component scores can reach comparable total scores. Fifthly, the imbalance in the distribution of HBV infection between the cases and controls might influence the association between the two indexes (CHEI/HEI-2015) and PLC risk. However, HBV infection seemed not to significantly modify the studied association (*p*-interactions > 0.05) and might slightly underestimate the studied association since HBV (+) subjects had slightly higher scores of CHEI (66 vs 64) and HEI-2015 (63 vs 61) in this study. Finally, residual confounding is still possible, despite adjustment for many potential confounders. 

## 5. Conclusions

Our findings suggest that adhering to the most recent dietary guidelines for the Chinese or Americans may reduce the risk of PLC. Well-designed perspective cohort studies and randomized clinical trials are needed to confirm these results. 

## Figures and Tables

**Table 1:** Characteristics of cases of primary liver cancer and the corresponding controls.

	Cases (*n* = 720)	Controls (*n* = 720)	*p-*Value
Age (year)			
Mean	58.2 ± 8.8	58.4 ± 8.1	0.718 ^*^
Sex, *n* (%)			
Women	107 (14.9)	107 (14.9)	1.000 ^+^
Men	613 (85.1)	613 (85.1)	
Body mass index (kg/m^2^) ^1^	22.8 ± 3.2	23.7 ± 3.1	<0.001 ^*^
Physical activity (MET-h/d) ^1^	29.3 (25.4, 38.8)	31.8 (25.6, 48.5)	<0.001 ^#^
Marital status, married, *n* (%)	701 (97.4)	676 (93.9)	0.001 ^+^
Education level, *n* (%)			
Secondary school or below	382 (53.1)	214 (29.7)	<0.001 ^+^
High school or above	338 (46.9)	506 (70.3)	
Household monthly income per capita, *n* (%)			
≤¥1500	110 (15.3)	207 (28.8)	<0.001 ^+^
¥1501~3000	195 (27.1)	300 (41.7)	
≥¥3001	415 (57.6)	213 (29.6)	
Smoker status, *n* (%)	412 (56.7)	356 (49.0)	0.003 ^+^
Never	307 (42.6)	380 (52.9)	
Former	98 (13.6)	65 (9.1)	
Current	315 (43.8)	273 (38.0)	
Alcohol consumption, *n* (%)			<0.001 ^+^
No Intake (0 g/day)	240 (33.3)	116 (16.1)	
Low Intake (1–15 g/day)	97 (13.5)	69 (9.6)	
High Intake (>15 g/day)	137 (19.0)	42 (5.8)	
Tea drinking, *n* (%)	436 (60.6)	469 (65.1)	0.077
History of diabetes, *n* (%)	76 (10.6)	57 (7.9)	0.212
HBV infection, *n* (%) ^2^	627 (87.6)	87 (12.2)	<0.001 ^+^
HCC, *n* (%)	676 (93.6)	-	
CHEI ^1^	51.92 ± 10.31	63.95 ± 7.58	<0.001 ^*^
HEI-2015 ^1^	56.93 ± 6.68	61.43 ± 5.60	<0.001 ^*^
Total Energy (kcal/day) ^1,^^3^	2021.01 ± 615.52	2103.02 ± 662.11	0.015 ^*^

MET-h/day, Metabolic equivalent hours per day; HBV, Hepatitis B virus; HCC, Hepatocellular carcinoma; CHEI, Chinese Healthy Eating Index; HEI-2015, Healthy Eating Index 2015. ^1^ Values are mean ± SD or median (P25, P75), where appropriate. ^2^ HBV infection was defined as HBV surface antigen positivity. ^3^ Total Energy intake was dietary energy except for alcohol. ^*^ *p*-values, two-sided student’s *t* test for the normally distributed continuous variables. ^#^ *p*-values, two-sided Wilcoxon rank-sum test for the skewed distributed continuous variables. ^+^ *p*-values, two-sided chi-square test for the categorical variables.

**Table 2:** ORs (95% CIs) for primary liver cancer per 5-point increment of diet-index scores stratified by selected factors.

	Crude		Age- and Sex-Adjusted		Multivariable-Adjusted ^1^		*p-*Interaction
	OR	95% CI	OR	95% CI	OR	95% CI	
**CHEI**							
**Total scores**	0.47	0.44, 0.51	0.47	0.44, 0.51	0.43	0.38, 0.50	
**Sex**				.			0.609
Women	0.61	0.51, 0.73	0.61	0.51, 0.73	0.55	0.43, 0.71	
Men	0.45	0.41, 0.49	0.48	0.41, 0.49	0.40	0.34, 0.47	
**Smoker** **status**							0.810
Never	0.51	0.45, 0.57	0.51	0.45, 0.57	0.45	0.37, 0.55	
Former	0.45	0.34, 0.59	0.45	0.34, 0.59	0.41	0.25, 0.67	
Current	0.42	0.37, 0.49	0.42	0.37, 0.48	0.41	0.32, 0.51	
**Alcohol Consumption**							0.850
No Intake	0.48	0.44, 0.53	0.48	0.44, 0.53	0.43	0.37, 0.50	
Low Intake	0.43	0.33, 0.56	0.43	0.33, 0.56	0.32	0.17, 0.58	
High Intake	0.55	0.44, 0.68	0.55	0.44, 0.68	0.37	0.21, 0.66	
**HBV infection**							0.686
Yes	0.45	0.38, 0.53	0.45	0.38, 0.53	0.42	0.34, 0.51	
No	0.45	0.39, 0.53	0.45	0.38, 0.53	0.46	0.37, 0.54	
**HCC**							-
Yes	0.47	0.44, 0.51	0.47	0.44, 0.52	0.42	0.37,0.49	
No	0.44	0.36, 0.54	0.45	0.36, 0.54	0.41	0.31, 0.53	
**HEI-2015**							
**Total scores**	0.55	0.50, 0.61	0.55	0.50, 0.60	0.47	0.40, 0.55	
**Sex**							0.583
Women	0.65	0.53, 0.80	0.65	0.52, 0.80	0.55	0.39, 0.76	
Men	0.53	0.47, 0.59	0.53	0.48, 0.59	0.46	0.38, 0.55	
**Smoker** **status**							0.704
Never	0.61	0.54,0.70	0.61	0.53, 0.70	0.53	0.42, 0.66	
Former	0.53	0.40, 0.72	0.53	0.39, 0.71	0.52	0.31, 0.86	
Current	0.49	0.42, 0.58	0.49	0.42, 0.58	0.40	0.30, 0.53	
**Alcohol consumption**							0.533
No Intake	0.58	0.52, 0.65	0.57	0.51, 0.64	0.47	0.39, 0.56	
Low Intake	0.42	0.30, 0.59	0.42	0.30, 0.60	0.34	0.16, 0.71	
High Intake	0.57	0.43, 0.76	0.58	0.43, 0.77	0.55	0.33, 0.91	
**HBV infection**							0.552
Yes	0.49	0.40, 0.59	0.50	0.41, 0.61	0.45	0.36, 0.57	
No	0.49	0.40, 0.61	0.49	0.40, 0.61	0.51	0.41, 0.65	
**HCC**							-
Yes	0.56	0.51, 0.61	0.55	0.50, 0.61	0.46	0.39, 0.55	
No	0.44	0.31, 0.63	0.44	0.31, 0.63	0.44	0.32, 0.62	

Abbreviations: CHEI, Chinese Healthy Eating Index; HEI-2015, Healthy Eating Index 2015; HBV infection, Hepatitis B virus infection; HCC, Hepatocellular carcinoma; ORs, Odds ratios. ^1^ Adjusted for age, sex, body mass index, physical activity, education, household income, smoker status, alcohol consumption, diabetes, HBV infection, and total energy.

**Table 3:** Association of each component score of CHEI and HEI-2015 with primary liver cancer risk between case and control.

	Maximum Score	Criteria for Maximum Score ^1^	Criteria for Minimum Score	OR ^2^	95% CI
**CHEI**					
Total Grains	5	≥2.5 SP /1000 Kcal	No Total Grains	1.15	0.87, 1.53
Whole Gains and Mixed Beans	5	≥0.6 SP/1000 kcal	No Whole Grains and Mixed Beans	0.60	0.51, 0.70
Tubers	5	≥0.3 SP/1000 kcal	No Tubers	1.13	1.00, 1.28
Total Vegetables	5	≥1.9 SP/1000 kcal	No Vegetables	0.82	0.71, 0.96
Dark Vegetables	5	≥0.9 SP/1000 kcal	No Dark Vegetables	0.87	0.75, 1.01
Fruits	10	≥1.1 SP/1000 kcal	No Fruit	1.09	1.03, 1.15
Dairy	5	≥0.5 SP/1000 kcal	No Dairy	0.54	0.48, 0.61
Soybeans	5	≥0.4 SP/1000 kcal	No Soybeans	0.86	0.78, 0.96
Fish and Seafood	5	≥0.6 SP/1000 kcal	No Fish and Seafood	0.68	0.61, 0.75
Poultry	5	≥0.3 SP/1000 kcal	No Poultry	0.50	0.44, 0.56
Eggs	5	≥0.5 SP/1000 kcal	No Eggs	0.61	0.54, 0.68
Seeds and Nuts	5	≥0.4 SP/1000 kcal	No Seeds and Nuts	0.64	0.59, 0.70
Red Meat	5	≤0.4 SP/1000 kcal	≥3.5 SP/1000 kcal	1.00	0.88, 1.14
Cooking Oil	10	≤15.6 g/1000 kcal	≥32.6 g/1000 kcal	0.71	0.65, 0.77
Sodium	10	≤1000 mg/1000 kcal	≥3608 mg/1000 kcal	0.69	0.64, 0.75
Alcohol	5	25 g (men)/15 g (women)	≥60 g (men)/40 g (women)	0.73	0.62, 0.85
**HEI-2015**					
Total Fruits	5	≥0.8 cup eq./1000 kcal	No Fruit	1.25	1.11, 1.41
Whole Fruits	5	≥0.4 cup eq./1000 kcal	No Whole Fruit	1.11	1.00, 1.23
Total Vegetables	5	≥1.1 cup eq./1000 kcal	No Vegetables	0.66	0.53, 0.83
Greens and Beans	5	≥0.2 cup eq./1000 kcal	No Dark Green Vegetables or Legumes	0.59	0.41, 0.86
Whole Grains	10	≥1.5 oz eq./1000 kcal	No Whole Grains	0.66	0.59, 0.74
Dairy	10	≥1.3 cup eq./1000 kcal	No Dairy	0.52	0.45, 0.61
Total Protein Foods	5	≥2.5 oz eq./1000 kcal	No Protein Foods	0.37	0.29, 0.47
Seafood and Plant Proteins	5	≥0.8 oz eq./1000 kcal	No Seafood or Plant Proteins	0.49	0.43, 0.57
Refined Grains	10	≤1.8 oz eq./1000 kcal	≥4.3 oz eq./1000 kcal	0.98	0.89, 1.07
Fatty Acids	10	(PUFAs + MUFAs)/SFAs ≥ 2.5 ^3^	(PUFAs + MUFAs)/SFAs ≤ 1.2	1.33	1.20, 1.47
Sodium	10	≤1.1g/1000 kcal	≥2.0g/1000 kcal	0.69	0.64, 0.75

^1^ Each component was awarded proportionately within the criteria.^2^ OR are adjusted for age, sex, body mass index, physical activity, education, household income, smoker status, alcohol consumption, diabetes, HBV infection, total energy. ^3^ A ratio of total unsaturated fatty acids (poly- and monounsaturated fatty acids [PUFAs and MUFAs]) to saturated fatty acids (SFAs).

## Supplementary Materials

The following are available online at http://www.mdpi.com/2072-6643/10/8/1113/s1, Table S1: Components of the scoring standards for each component of the CHEI and HEI-2015 by using standardized portion size.
