# Supplementary material for: Adherence to the Chinese or American Dietary Guidelines is Associated with a Lower Risk of Primary Liver Cancer in China: A Case-Control Study
Source: Nutrients. 2018 Aug 17;10(8):1113. doi: 10.3390/nu10081113 (PMC6115710; doi:10.3390/nu10081113)
Supplement: Supplementary file 1 [file nutrients-10-01113-s001.pdf]

Adherence to the Chinese or American Dietary Guidelines is Associated with a Lower Risk of Primary Liver Cancer in China: A Case-Control Study

Supplemental Table S1. Components of the scoring standards for each component of the CHEI and HEI-2015 by using standardized portion size [1].

	Dietary indexes, scoring and criteria [2]	
	CHEI	HEI-2015 [3]
Total Score	100	100
Component	17 components (5-10 points each)	13 components (5–10 each)
Vegetables	Total vegetables: ≥1.9 SP (5 points); Dark Vegetables: ≥0.9 SP (5 points); Tubers: ≥0.3 SP (5 points)	Total vegetables: ≥1.1 cup equivalents (5 points); greens and beans: ≥0.2 cup equivalents (5 points)
Fruit	Fruits: ≥1.1 SP (10 points)	Total fruits [4]: ≥0.8 cup equivalents (5 points); whole fruits [5]: ≥0.4 cup equivalents (5 points)
Nuts	Seeds and Nuts: ≥0.4 SP (5 points)	
Legumes	Whole grains and mixed beans: ≥0.6 SP (5 points)	Allocated to four components: Total protein foods, seafood and plant proteins, total vegetables and greens and beans.
Whole grains	-	Whole grains: ≥1.5 oz. equivalents (10 points)
Fish	Fish and Seafood: ≥0.6 SP (5 points)	Seafood and plant proteins [6]: ≥0.8 oz. equivalents (5 points)
Total protein foods	Soybeans: ≥0.4 SP (5 points); Poultry: ≥0.3 SP (5 points); Eggs: ≥0.5 SP (5 points); Red meat: ≥0.4 SP (5 points)	Total proteins foods: ≥2.5 oz. equivalents (5 points)
Dairy	≥0.5 SP (5 points)	≥1.3 cup equivalents (10 points)
Oils or fats	Cooking oils: ≤15.6 g (10 points)	(PUFAs + MUFAs)/SFAs ratios ≥2.5 (10 points) [8]; saturated fats: ≤8% of energy (10 points);
Alcohol	Alcohol: 25-60 g/d (men); 15-40 g/day (women) (5 points)	contribute calories to the total energy
Refined grains	Total grains [7]: ≥2.5 SP (5 points)	≤1.8 oz. equivalents (10 points)
Sodium	≤1000 mg (10 points)	≤1100 mg (10 points)
Added Sugars	≤10% of energy (5 points)	≤ 6.5% of energy (10 points)

CHEI, Chinese Eating Index; HEI-2015, Healthy Eating Index-2015. [1] Standardized portion sizes (SP) are based on the Dietary Guidelines for the Chinese, 2016. [2] All components are based on the density standard (per 1,000 kcal or percentage of energy), except for alcohol in the CHEI and fatty acid ratios in HEI-2015. [3] Components standardized in the HEI-2015 are based on the Food Patterns Equivalents Database (FPED), which was previously called the MyPyramid

Equivalents Database (MPED). [4] Total fruits included 100% fruit juice. [5] Whole fruits included all fruits except juice. [6] Seafood and plant proteins included seafood, nuts, seeds, soy products, and legumes. [7] Total grains included refined grains and whole grains. [8] A ratio of total unsaturated fatty acids (poly- and monounsaturated fatty acids [PUFAs and MUFAs]) to saturated fatty acids (SFAs).
